# Discovering and Creating the Leading Edge of Extended Reality and Spatial Computing: A Message From the Editor-in-Chief

**DOI:** 10.2196/64545

**Published:** 2024-07-26

**Authors:** Lars Riedemann

**Affiliations:** 1 Department of Neurology Heidelberg University Hospital Heidelberg Germany

**Keywords:** editorial, extended reality, XR, spatial computing

## Abstract

We are pleased to introduce *JMIR XR and Spatial Computing*, a peer-reviewed journal dedicated to advancing the integration of extended reality and spatial computing technologies into routine clinical care.

We are excited to announce the launch of *JMIR XR and Spatial Computing*, a peer-reviewed journal dedicated to showcasing research on extended reality (XR) and spatial computing technologies and their integration into everyday clinical practice.

In navigating the frontier of XR and spatial computing for more than a decade, we have adopted a stance of informed optimism tempered by vigilant caution. Based on the large number of high-quality articles published by JMIR Publications and others over the last decades, the tremendous potential of these technologies to enhance diagnostic precision, increase treatment efficacy, facilitate easier access to care, and—most importantly—improve patient outcomes seem obvious. Yet, we remain cognizant that their integration into the health care ecosystem is not without peril and may take longer than many researchers and technologists have expected. We anticipate that immersive technologies will transition from a novel solution to an established standard in targeted medical scenarios. However, integration into complex health care systems and widespread acceptance by health care workers will not happen overnight. Not unlike other established technologies, we expect a slow and stepwise adoption as a result of candid discussions within the community, and rigorous, basic, translational, and clinical research [1].

XR and spatial computing are relatively old concepts, dating back to at least the late 1960s; they became visible to the scientific community through iconic works such as Ivan Sutherland’s *The Sword of Damocles* [2]. In 2003, Simon Greenwold at the Massachusetts Institute of Technology described “spatial computing” in his thesis as a “human interaction with a machine in which the machine retains and manipulates referents to real objects and spaces” [3]. Inspired by prior researchers and technologists, he foresaw that “the augmentation of a data network with a physical network promotes the flow of digital information on top of existing social interactions” [3].

When JMIR Publications launched in 1999, the widespread adoption of XR and spatial computing in clinical settings was hard to imagine. Fast forward to today, and we find ourselves at the cusp of an era where XR and spatial computing are poised for integration into routine clinical care. This shift has been propelled by a confluence of recent technological advancements. The rapid artificial intelligence (AI) evolution [4], particularly breakthroughs in computer vision, has significantly improved spatial mapping and 3D scene understanding. Furthermore, AI has truly revolutionized programming code and 3D content creation. Exponential improvements in graphical processing capabilities [5] have been complemented by significant advances in lightweight, energy-efficient display technologies [6], and the advent of high-bandwidth and low-latency networks has significantly enhanced connectivity. The decreasing costs of XR technology and its growing popularity among consumers continue to lower adoption barriers for XR and spatial computing in health care.

The tangible impact of these developments in health care is evident. For instance, Bandelow et al [7], who authored the German guideline for treating anxiety disorders, recommend virtual reality exposure therapy as a viable alternative when in vivo exposure is not feasible for patients with spider, height, or flight phobias. Furthermore, the US Food and Drug Administration has reviewed and authorized the marketing of a growing number of devices with augmented reality and virtual reality through 510(k) clearance, De Novo requests, or premarket approval in many fields of medicine [8].

These examples illustrate the growing acceptance of immersive technologies in clinical practice as “another arrow in the quiver” of health care.

However, our optimism is tempered with pragmatism. Although XR and spatial computing offer promising avenues for enhancing health care delivery, we recognize that they are not universal solutions. The successful integration of these technologies into existing health care systems and workflows requires thoughtful consideration and careful implementation [8]. Their true effectiveness will be determined by the appropriateness of their application, the specific contexts in which they are deployed, a significantly positive cost-benefit ratio, and most importantly, their demonstrable ability to improve patient outcomes or enhance health care efficiency [8,9]. As we move forward, it is crucial to approach the adoption of these technologies with a balanced perspective, ensuring that their integration complements and enhances, rather than disrupts, the foundational aspects of quality health care delivery.

Therefore, we encourage authors from both academia and industry to view *JMIR XR and Spatial Computing* as a platform for showcasing their collaborative efforts, sharing insights, and contributing to the responsible advancement of immersive technologies in health care.

Our journal recognizes the critical importance of addressing the accessibility and equity challenges surrounding XR and spatial computing technologies. We strongly encourage the community to submit manuscripts exploring innovative solutions to these pressing issues. We are particularly interested in studies on cost-effective technology implementations in resource-limited settings and strategies for overcoming infrastructure barriers in underserved areas. By fostering dialogue and research in this area, we aim to ensure that the transformative potential of XR and spatial computing technologies benefits the entire international community.

Central to our vision is the belief in the power of strong academic-industrial collaborations. These collaborations bridge the gap between theoretical research and practical application, accelerating the development of cutting-edge XR and spatial computing solutions while ensuring they meet rigorous scientific standards. Therefore, we actively encourage and facilitate partnerships between academic researchers and industry innovators. Furthermore, we invite independent, nonacademic developers and designers as well as open-source project contributors of all kinds to communicate their perspectives with us. We believe that the transparency and accessibility of open-source development can significantly accelerate progress in XR and spatial computing applications for health care.

By embracing contributions from this diverse range of sources—academic-industrial collaborations, individual innovators, and open-source communities—we aim to foster a rich ecosystem of ideas and developments that will shape the future of XR and spatial computing in health care.

## Abbreviations

AI: artificial intelligence
XR: extended reality
